# Gene expression variability across cells and species shapes the relationship between renal resident macrophages and infiltrated macrophages

**DOI:** 10.1186/s12859-023-05198-z

**Published:** 2023-03-01

**Authors:** Xiangjun Ji, Junwei Cai, Lixin Liang, Tieliu Shi, Jinghua Liu

**Affiliations:** 1 Guangdong Provincial Key Laboratory of Proteomics, Department of Pathophysiology, School of Basic Medical Sciences, Southern Medical University, Guangzhou, 510515 China; 2 Center for Bioinformatics and Computational Biology, The Institute of Biomedical Sciences and School of Life Sciences, East China Normal University, Shanghai, 200241 China; 3 Beijing Advanced Innovation Center, for Big Data-Based Precision Medicine, Beihang University and Capital Medical University, Beijing, 100083 China

**Keywords:** Renal macrophage, Evolutionary conservation, Transcriptional divergence, Signaling transduction, Gene regulatory network (GRN)

## Abstract

**Background:**

Two main subclasses of macrophages are found in almost all solid tissues: embryo-derived resident tissue macrophages and bone marrow-derived infiltrated macrophages. These macrophage subtypes show transcriptional and functional divergence, and the programs that have shaped the evolution of renal macrophages and related signaling pathways remain poorly understood. To clarify these processes, we performed data analysis based on single-cell transcriptional profiling of renal tissue-resident and infiltrated macrophages in human, mouse and rat.

**Results:**

In this study, we (i) characterized the transcriptional divergence among species and (ii) illustrated variability in expression among cells of each subtype and (iii) compared the gene regulation network and (iv) ligand-receptor pairs in human and mouse. Using single-cell transcriptomics, we mapped the promoter architecture during homeostasis.

**Conclusions:**

Transcriptionally divergent genes, such as the differentially TF-encoding genes expressed in resident and infiltrated macrophages across the three species, vary among cells and include distinct promoter structures. The gene regulatory network in infiltrated macrophages shows comparatively better species-wide consistency than resident macrophages. The conserved transcriptional gene regulatory network in infiltrated macrophages among species is uniquely enriched in pathways related to kinases, and TFs associated with largely conserved regulons among species are uniquely enriched in kinase-related pathways.

**Supplementary Information:**

The online version contains supplementary material available at 10.1186/s12859-023-05198-z.

## Background

Innate immunity is the first line of host defense against infection and is critical for the development of adaptive immunity [1]. Macrophages are effector cells of the innate immune system [2]. In nearly all solid tissues, two main subsets of macrophages are evident: resident and infiltrated macrophages [3]. Resident macrophages arising from the embryos reside in tissues under both homeostatic and inflammatory conditions [4, 5]. They exhibit phagocytic functions, mediate the inflammatory response and regulate tissue repair [6, 7]. In contrast, recruited infiltrated macrophages arising from circulating monocytes display high production of both pro-inflammatory cytokines and chemokines that induce inflammation [3]. Recent evidence suggests that macrophage programming is multidimensional, dynamic, and exceedingly complex at the individual level [8]. In addition, many genes related to the innate immune response have rapidly evolved in the vertebrate lineage [9, 10]. Notably, single-cell RNA sequencing (scRNA-seq) is a unique method used to gain insight into the transcriptome of renal macrophages at the single-cell level [11–14]. A recent study on innate immune cells in the kidney identified universal markers for detecting renal resident macrophages among human, mouse, and rat based on scRNA-seq and flow cytometry. This original analysis provided pre-processed scRNA-seq data that we have analyzed [7].

In this study, we aimed to identify heteogeneity among renal macrophages as determined at single-cell resolution across species. We wondered whether there are connections among (1) cross-species transcriptional divergence of differentially expressed genes between resident and infiltrated macrophages; (2) the promoter architecture; and (3) cell-to-cell variability. In our paper, we found that genes with highly divergent expression among the three species exhibited higher cell-to-cell variability than genes with little expression divergence (see “Methods”). We also wondered whether the mechanism of transcriptional regulation can explain the difference in renal macrophages from different species and different subtypes. We explored the transcriptional signatures of resident and infiltrated macrophages in human, mouse, and rat during homeostasis. Furthermore, we characterized the activity of the gene regulatory network and role played by cellular crosstalk since they are crucial for understanding the mechanism of gene expression regulation.

## Results

### Transcriptional divergence between resident and infiltrated macrophages in the kidney

**Fig. 1:**
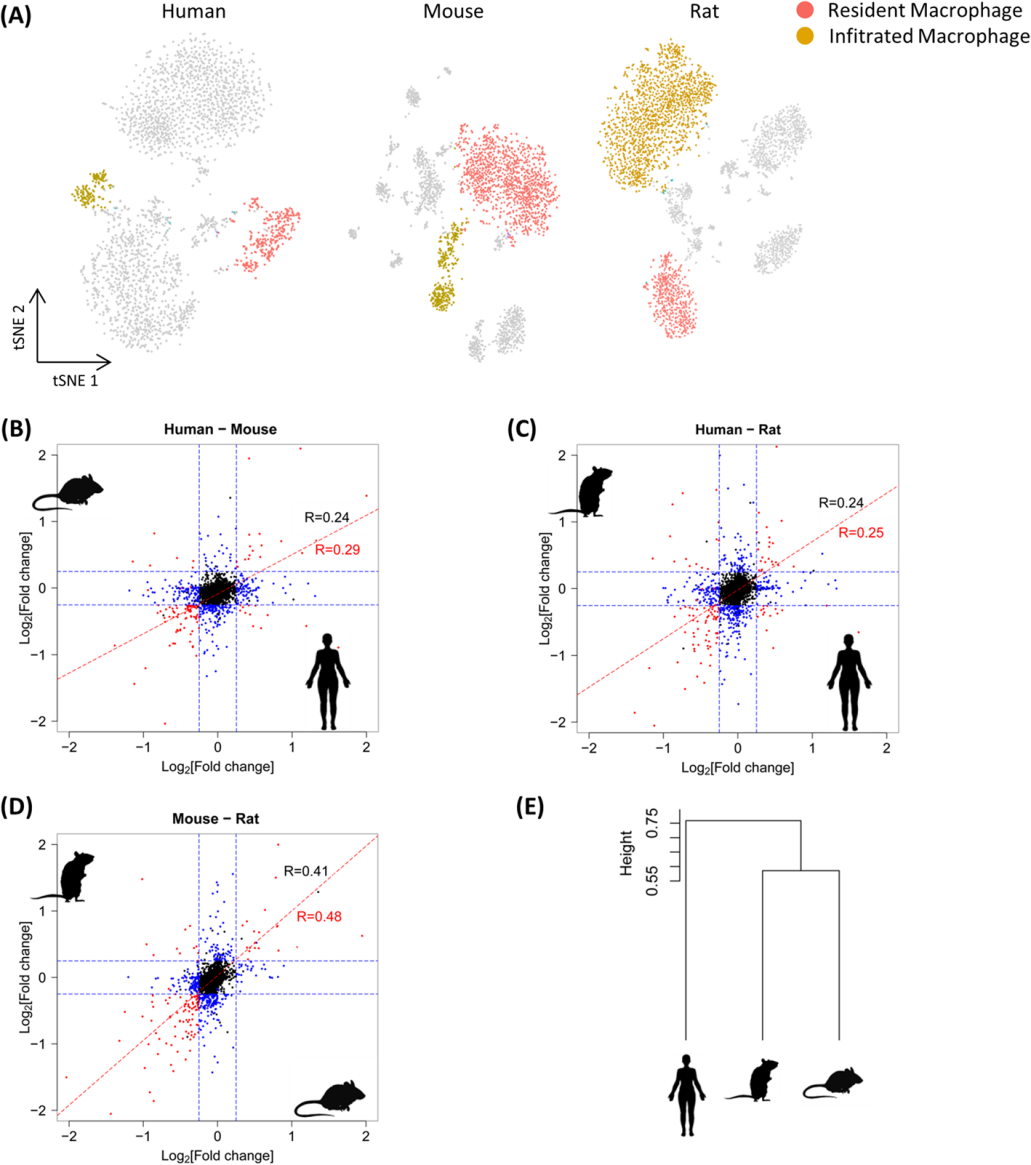
**A** t-SNE plot showing manually annotated human, mouse, and rat renal macrophages. **B**–**D** Transcriptional divergence in resident and infiltrated macrophages between species. Fold-change values of all expressed genes (with one-to-one orthologs) between resident and infiltrated macrophages in pairs of species (human-mouse, mouse–rat and human–rat pairs). Spearman correlations between all expressed one-to-one orthologues are shown in black, and Spearman correlations between the subset of DEGs (FDR-adjusted *P-*value < 0.05 in both species) appear in red. Genes that are differentially expressed (FDR-adjusted *P-*value < 0.05) in both species and corresponding spearman correlation coefficients are in red, differentially expressed in only one species are in blue, and genes that are not differentially expressed are in black. Spearman correlation coefficients of all genes are in black. **E** Dendrogram based on the fold-change between resident and infiltrated macrophages of all expressed genes across one-to-one orthologs in human, mouse and rat

Using the aforementioned preprocessed scRNA-seq data [7], we reanalyzed 2868, 3013, and 3935 innate immune cells from human, mouse, and rat kidney tissue, respectively, that had been identified based on strict quality control thresholds (Fig. 1A), and the results were consistent with the results reported in [7]. For the current analysis, we labeled resident and infiltrated macrophages while excluding other types of cells on the basis of their expression pattern (CD74 and CD81 are markers of resident macrophages; S100A8^−^IRF7^+^ and S100A8^−^CEBPB^+^ cells were infiltrated macrophages, as reported in [7]). We generated a cell-specific gene set scoring system, and the results showed that resident macrophages could be distinguished from infiltrated macrophages in all species on the basis of their antigen processing and presentation (FDR-adjusted *P-*value < 0.05).

Some identical differentially expressed genes (DEGs) are known markers of resident macrophages in the kidney (e.g., CD74 and CD81) among species [7]. Upregulated DEGs in resident macrophages were enriched in pathways related to classical functions of macrophages; e.g., they were expressed in ribosomes and lysosomes (FDR-adjusted, *P-*value < 0.05) [15] (Additional file 2: Table S4). This is coupled to a form of autophagy to maintain homeostasis termed lysosome-associated phagocytosis (LAP) in which part of the autophagy machinery is engaged to clear the apoptotic material while at the same time induce the production of the tolerance-inducing cytokines IL-10 and TGF-β that prevent an immune response [16, 17]. In contrast, upregulated DEGs in infiltrated macrophages were enriched in pathways related to the production of pro-inflammatory cytokines, e.g., the chemokine signaling pathway, Rap1 signaling pathway and MAPK signaling pathway (FDR-adjusted, *P-*value < 0.05) (Additional file 2: Table S5). Collectively, these data reveal the consistency of the functional differences between resident and infiltrated macrophages in the three species.

To study the similarity of gene expression divergence in resident and infiltrated macrophages across the three species, we performed a correlation analysis based on orthologous gene expression. We plotted the fold-change values of all expressed genes with one-to-one orthologs between pairs of species (human–mouse, mouse–rat and human–rat pairs) (Fig. 1B–D). We observed that fold-change estimates between species were correlated (Spearman correlation, P < 10^−10^, in all comparisons) [18]. Furthermore, the divergence in gene expression in resident and infiltrated macrophages tended to be more strongly correlated between closely related species (mouse–rat pairs) than between more distantly related species (human–mouse and human–rat pairs), as shown in other expression programs (Fig. 1E) [19]. Finally, we identified 294, 278, and 350 differentially expressed genes between resident and infiltrated macrophages across human, mouse, and rat, respectively (FDR-adjusted P value < 0.05) using one-to-one orthologue mapping (Additional file 2: Table S1–S3). The correlation coefficients of the DEGs were higher than those of all genes in all pairs, which implied that DEGs between resident and infiltrated macrophages were more highly evolutionary conserved than the other genes. We classified the 636 DEGs into three groups on the basis of their levels of transcriptional divergence to tell the difference of genes with different evolutionary consistency measured by fold changes between infiltrated and resident macrophages in each species (see “Methods”). There was significant difference between transcriptional divergence values from different groups (one-sided Mann‒Whitney test, *P-*value < 0.01) (Additional file 1: Fig. S1).

### Promoter architecture of diverging genes

**Fig. 2:**
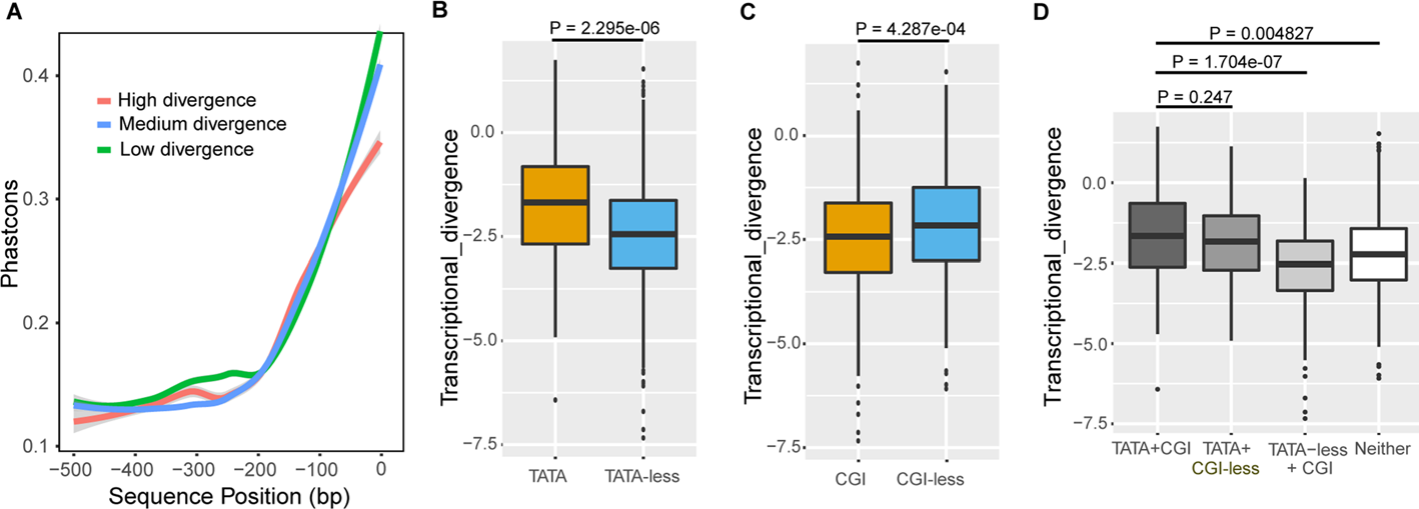
DEGs with different transcriptional divergence have different promoter architectures. **A** Promoter sequence conservation and transcriptional divergence in resident and infiltrated macrophages among human, mouse, and rat. Mean conservation values of each of the 500 bps upstream of the TSS are shown for high-, medium- and low-divergence genes (n = 636 DEGs). The 95% confidence interval for predictions is shown in grey. **B** Comparison of transcriptional divergence of genes with and without a TATA-box (one-sided Mann‒Whitney test, *P-*value < 0.01). **C** Comparison of transcriptional divergence of genes with and without a CGI (one-sided Mann‒Whitney test, *P-*value < 0.01). **D** Comparison of transcriptional divergence of genes with and without a TATA-box and a CGI (one-sided Mann‒Whitney test, *P-*value < 0.01)

To quantify cross-species transcriptional divergence between resident and infiltrated macrophages, we focused on 636 DEGs with one-to-one orthologues across all three species in the kidney (see “Methods”, Additional file 2: Table S6). We tested whether cross-species divergence between resident and infiltrated macrophages is reflected in the conservation of promoter function and/or sequence. Promoters enriched with TATA-boxes and depleted of CGIs are thought to be highly conserved. First, we compared the conservation of sequences 500 bp upstream of the TSS in high- versus both medium- and low- divergent human genes (see “Methods”). Genes with high divergence showed lower sequence conservation in the 500 bp upstream of the TSS than genes with both medium divergence and low divergence (one-sided Kolmogorov–Smirnov test, *P-*value < 0.01), which was consistent with current thinking (Fig. 2A) (see “Methods”). Notably, when the TATA-box in the core promoter region (100 bp upstream of the transcription start site) is taken into account [20], genes with highly divergent expression and TATA-box promoters tended to evolve significantly slower than genes with highly divergent expression and no TATA-box promoter (one-sided Kolmogorov–Smirnov test, *P-*value < 0.01), as reported for other immune contexts [21]. Furthermore, the proportion of highly–divergent genes with TATA-box promoters was significantly higher than the proportion of low–divergent genes with TATA-box promoters (Fisher's exact test, *P-*value < 0.01), which may have been related to promoters in genes with highly and low-divergent expression showing distinctive architectures [22–24].

Second, we found that genes with TATA-boxes were associated with higher transcriptional divergence (one-sided Mann‒Whitney test, *P-*value < 0.01) (Fig. 2B), while genes with CpG islands (CGIs) diverged more slowly than genes without CGIs (one-sided Mann‒Whitney test, *P-*value < 0.01) (Fig. 2C). Genes with a TATA-box but without a CGI show no significant difference in transcriptional divergence compared to genes with both elements; genes with a CGI but without a TATA-box diverged more slowly than genes with both elements. These results illustrate that TATA-boxes exert a stronger influence on transcriptional divergence than CGIs (Fig. 2D).

To investigate whether different functional classes among DEGs are characterized by significantly different scores of transcriptional divergence, we divided the 636 DEGs into categories according to their biological function. Since chemokines belong to a family of cytokines, we classified cytokines, chemokines and their receptors as cytokines. We found that the divergence of DEGs encoding 18 cytokines (e.g., CCL3) and 26 TFs (e.g., JUNB) tended to be higher than that of other DEGs (one-sided Mann‒Whitney test, *P-*value = 0.0131 and 0.03415, respectively) (Additional file 1: Fig. S2), and promoters of TFs were enriched with TATA-boxes (Fisher's exact test, 34.62% versus 15.57%, *P-*value = 0.025), suggesting the versatile TF divergence among species.

### Cell-to-cell variability in renal macrophages

**Fig. 3:**
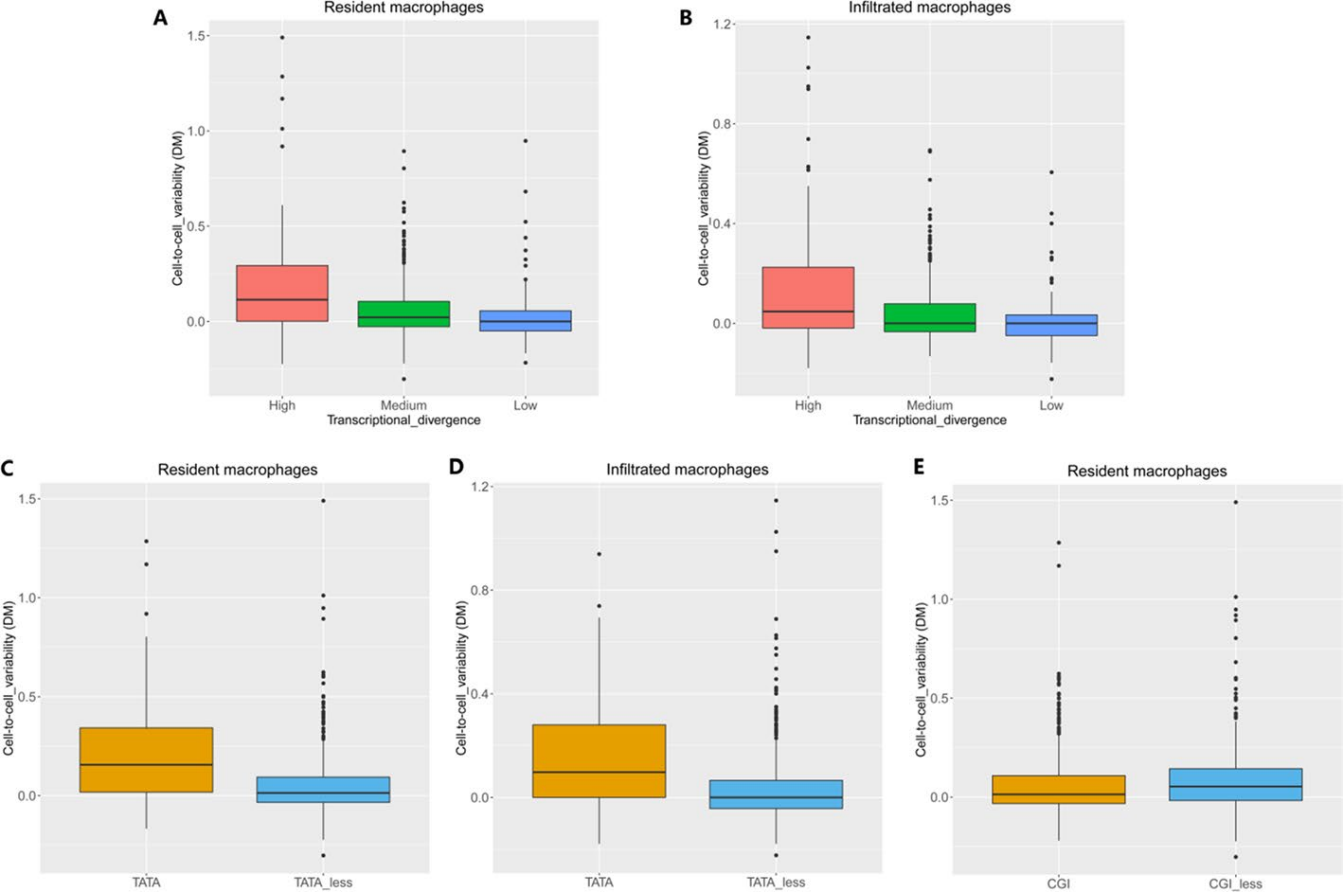
Cell-to-cell variability analysis of human renal macrophages. Comparison of transcriptional divergence across species with cell-to-cell variability between individual cellswhere the variability were measured in **A** n = 318 resident macrophages (high vs low, one-sided Mann‒Whitney test, *P-*value < 0.01) and **B** n = 143 infiltrated macrophages (high vs low, one-sided Mann‒Whitney test, *P-*value < 0.01) in human kidney. Comparison of cell-to-cell variability of genes with and without a TATA-box in **C** resident macrophages (one-sided Mann‒Whitney test, *P-*value < 0.01) and **D** infiltrated macrophages (one-sided Mann‒Whitney test, *P-*value < 0.01). **E** Comparison of cell-to-cell variability of genes with and without a CGI in resident macrophages (one-sided Mann‒Whitney test, *P-*value = 0.0179)

To study heterogeneity in gene expression across individual cells of a certain subtype, we quantitatively estimated cell-to-cell variability using an established measure for variability: distance to median (DM) (see “Methods”) [7]. Intriguingly, we found that genes with highly divergent expression among the three species showed higher cell-to-cell variability than low-divergence genes across individual cells in human (one-sided Mann‒Whitney test, *P-*value < 0.01) (Fig. 3A, B). Same results were for mouse and rat. Notably, the differences we observed were not caused by technical biases due to low expression levels in the scRNA-seq data (Additional file 1: Fig. S3).

Next, we examined the relationship between the presence of promoter elements (CGIs and TATA-boxes) and the cell-to-cell variability of genes. We found that genes predicted to have a TATA-box in their promoters in human showed higher transcriptional variability in both resident and infiltrated macrophages in human (one-sided Mann‒Whitney test, *P-*value < 0.01) (Fig. 3C, D), in agreement with previous findings [25]. Same results were observed in mouse and rat. CGI-containing genes showed higher transcriptional variability only in resident macrophages (one-sided Mann‒Whitney test, *P-*value = 0.0179) (Fig. 3E). Thus, both transcriptional variability between cells (Fig. 3C–E) and transcriptional divergence between species (Fig. 2B, C) were associated with specific promoter elements.

### Gene regulation network between resident and infiltrated macrophages in human and mouse kidneys

**Fig. 4:**
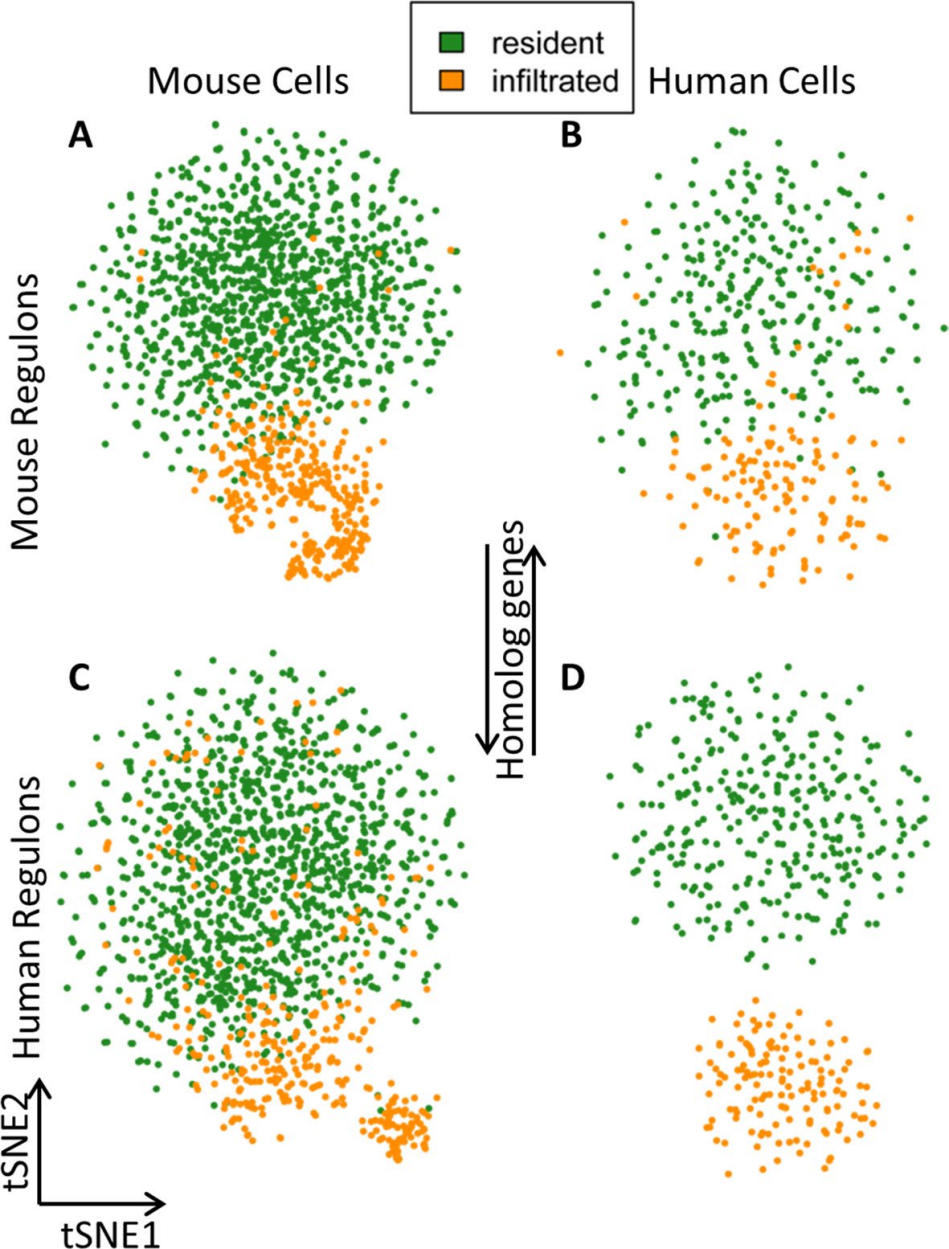
Reciprocal activity of human and mouse regulons on mouse and human scRNA-seq data. Each cell is assigned the color of a macrophage subtype based on the expression matrix inferred by Seurat. The regulon activity can be achieved with expression matrix and regulons from the same species (e.g. Fig. 4**A** and **D**), or from different species by converting TFs and the corresponding target genes into the other species (e.g. Fig. 4**B** and **C**)

The activity of co-expressed TF sub-networks or 'regulons' can be used to identify cell subsets [26, 27]. Therefore, the activity of TFs in a cell may be estimated partially through the expression of their putative targets [28]. We constructed regulons with our datasets for human and mouse respectively by employing pySCENIC [28–30]. Regulons of rat were not constructed because the relevant databases were not available for this species. Resident and infiltrated macrophages in each species were clearly distinguished based on distinct regulon activity among different cell types in the corresponding species (Fig. 4A, D). We analyzed the scRNA-seq dataset of human renal macrophages by exploiting regulons identified in mouse renal macrophages to validate the evolutionary conservation of mouse regulons and vice versa. The results showed that human and mouse regulons can be used reciprocally to distinguish the two subsets of macrophages in mouse and human to a large extent (Fig. 4B, C).

**Fig. 5:**
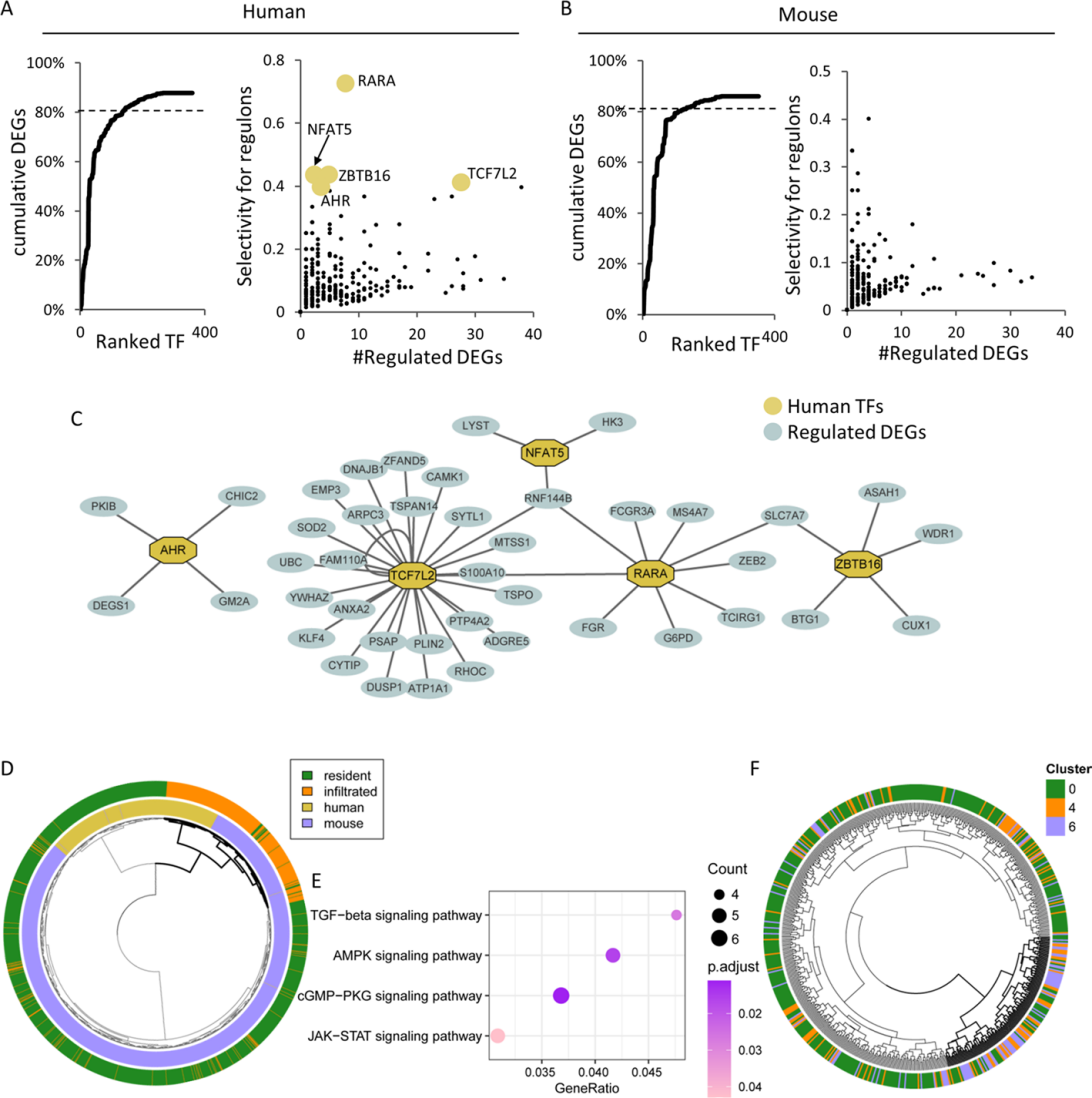
Comparative analysis of regulons between human and mouse. **A** and **B** Left: Cumulative percentage of regulated DEGs by TFs sorted on the basis of their selectivity. Right: scatterplot showing the number of DEGs regulated by TFs (only TFs regulating < 40 DEGs are shown) and the selectivity of gene regulation for DEGs. The 5 genes with the highest selectivity scores in human are highlighted. **C** Schematic illustration showing the regulons associated with the 5 TFs with the highest selectivity in human. **D** Joint clustering of human and mouse renal scRNA-seq data based on the activity of regulons with DEGs as target genes. The branch in which infiltrated macrophages showed a dominant position is shown in bold. **E** KEGG enrichment of TFs associated with regulons that showed greater evolutionary conservation in infiltrated macrophages. (F) Kidney cells from septic mouse analyzed at the 48-h time point in Dagher’s paper were clustered on the basis of the activity of all the regulons obtained in the original paper. The labels of the cells were based on the clusters described in Dagher’s paper. The branch that is composed mostly of Cluster 4 and Cluster 6 is shown in bold

We identified 360 and 350 regulons in human and mouse macrophages, respectively (Fig. 5A, B, Additional file 2: Table S7 and S8). We ranked the identified TFs by their selectivity for DEGs in resident and infiltrated macrophages (see “Methods”). We found that the top 134 TFs (of 360 TFs) in human and 106 TFs (of 350 TFs) in mouse regulated more than 80% of the DEGs (Fig. 5A, B), and only 39 of these TFs were common between the two species (Additional file 2: Tables S7 and S8). Among the 636 DEGs in the resident and infiltrated macrophages, 15 and 18 genes encode TFs in human and mouse regulons, respectively. The selectivities of the regulons composed of these differentially expressed TFs were significantly higher than the selectivities of regulons composed of other TFs in both human and mouse kidneys (one-sided Mann‒Whitney test, *P-*value < 0.01) (Additional file 2: Tables S7 and S8), which may be explained by the dependence of a regulon on the coexpression of a TF and its target gene. We also found that normalized binary entropy scores of regulons that were correlated with at least one other regulon (|r|> 0.3) were significantly lower than the scores of other regulons in both human and mouse kidney (one-sided Mann‒Whitney test, *P-*value < 0.01). These regulons alone were the perfect determinant for clustering resident and infiltrated macrophages in both human and mouse (Additional file 1: Fig. S6). Taken together, these results indicate that subtype-specific regulons were inclined to work synergistically to boost divergence between resident and infiltrated macrophages in both human and mouse kidneys. There were 18 such common regulon pairs in human and mouse, i.e., Fos and Jun (Additional file 2: Table S15). A large proportion of the DEGs (73.3% in human and 76.7% in mouse) were regulated by multiple TFs, and this proportion was significantly higher than that of non-DEGs in both human and mouse (proportion test, P < 0.05) (Fig. 5C, Additional file 2: Tables S7 and S8). The majority of TFs were controlled by other TFs (328 of 360 TFs in human and 297 of 350 TFs in mouse) (Additional file 2: Tables S7 and S8).

Joint clustering of human and mouse data based on both normalized expression and regulon activity yielded marked species-driven clustering (Additional file 1: Fig. S4). In contrast, the clustering analysis of regulons with selectivities greater than 0, i.e., regulons containing more than one DEG target genes, grouped both human and mouse infiltrated macrophages together (Fig. 5D). This finding suggests that regulons with selectivities greater than 0 were more robust than all the other regulons with respect to minimizing the species-driven batch effect. We then divided TFs into 2 groups according to comparative conservation of the corresponding regulon in infiltrated macrophages (see “Methods”): One group of TFs were more likely to be conserved in infiltrated macrophages between human and mouse, and others were less likely to be conserved. TFs associated with regulons highly conserved between species were uniquely enriched in pathways focused on kinases, e.g., the cGMP-PKG signaling pathway, AMPK signaling pathway [31], TGF-beta signaling pathway [32], JAK-STAT signaling pathway [33], etc. (Fig. 5E, Additional file 2: Tables S9 and S10)) [34]. This result was similar to a finding previously reported in which the author compared the transcriptional divergence as a difference in mononuclear phagocytes among species at different infection stages [35].

Identical regulons include those associate with TFs involved in classical immune functions and those with high normalized binary entropy scores (see “Methods”), e.g., NFKB1 and NFKB2. We found that inflammation-related regulons, e.g., NFKB2 and IRF7, were significantly enriched in infiltrated macrophages in both human and mouse (Fisher's exact test, FDR-adjusted *P-*value < 0.05), which is in accordance with the results of a previous study [3]. We observed a negative correlation between the normalized binary entropy score and selectivity of regulons (Pearson correlation r: −0.3 and −0.22 for human and mouse, respectively, *P-*value < 0.05). This finding suggests that a regulon in a higher proportion of differentially expressed target genes between human and mouse is likely to be specifically active in a certain type of macrophage in both human and mouse.

Finally, we asked whether our regulons constructed by mouse renal macrophages can be used to stratify the mouse renal macrophages described in Dagher’s paper [36]. Forty-eight hours after renal macrophages were treated with LPS are mostly categorized into 3 subtypes in Dagher’s paper: Cluster 0 (C0), Cluster 4 (C4) and Cluster 6 (C6). We chose to examine macrophages at the 48-h timepoint since it is within the recovery stage with respect to the physical condition of the mouse in Dagher’s paper and because we had obtained sufficient scRNA-seq data. Intriguingly, the use of our regulons enabled us to stratify Dagher’s data into two branches, C0 and C4C6 (Fig. 5F, see “Methods”). This bifurcation differs from the results presented Dagher’s paper, which divides the clusters into C0C4 (named Mφ-A in Dagher’s paper) and C6 (named Mφ-B in Dagher’s paper) on the basis of gene expression data. Although TFs associated with regulons that were preferably active in each respective branch were mostly enriched in common functions, TFs associated with regulons that are more active in the C4C6 cluster were uniquely enriched in cell cycle functions, similar to previously published results indicating that subsets of macrophages were involved in the regulation of the cell cycle (Additional file 2: Tables S11 and S12) [3, 37]. These TFs included Hinfp, Phf8, and three E2F family members: E2f1, E2f4, and E2f6. Regulons involved with E2f4 and Phf8 are found to be active in a significantly higher proportion of cells in the C4 cluster than in the C0 cluster (proportion test, P < 0.05). This result, coupled with the absence of proliferation markers in the C4 cluster, suggested that the C4 cluster represents an intermediate state between the C0 and C6 clusters with respect to cell replication. We then clustered renal macrophages at the 48 h time point in Dagher’s paper with regulons obtained through Dagher’s gene expression data. Although C6 diverged from the two other clusters, the C0 was cluster further divided into two segments (Additional file 1: Fig. S5).

### Cellular crosstalk between resident and infiltrated macrophages in human and mouse kidneys

**Fig. 6:**
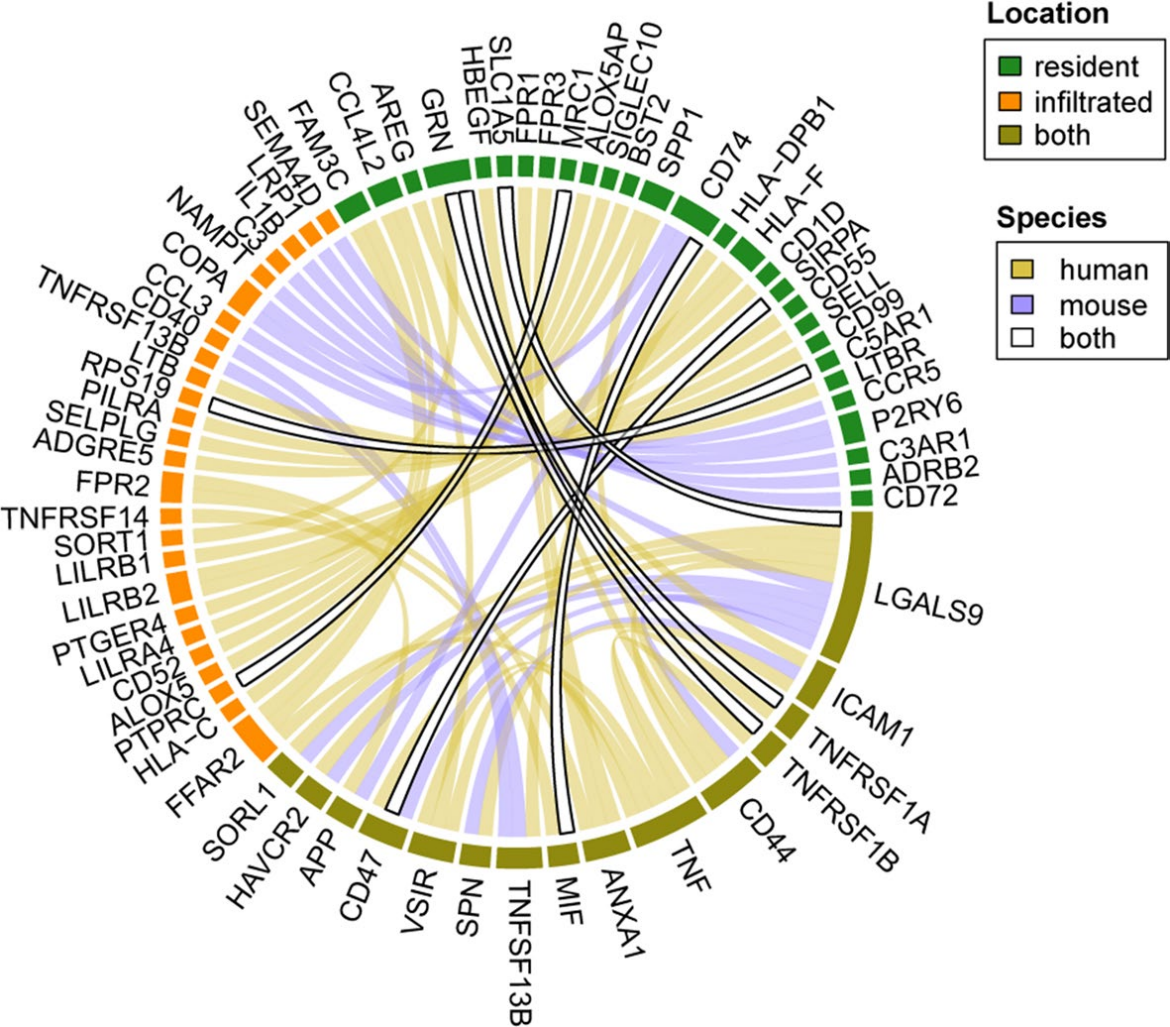
Cell‒cell signaling communication between resident and infiltrated renal macrophages in human and mouse. The 7 bands with a white background and black border indicate the common ligand‒receptor pairs in human and mouse. The color of each split ring indicates the type of macrophage in which a pair mediates signaling among species

We used CellPhoneDB [38] to infer cell–cell communication and found 41 and 23 ligand‒receptor interactions in resident and infiltrated macrophages in human and mouse, respectively, and 7 of these ligand–receptor pairs were common in the two species (Fig. 6, Additional file 2: Tables S13 and S14). Among these pairs, MIF-CD74 was identified, and CD74 is one of two universal markers (CD74 and CD81) of renal resident macrophages among species, as reported in aforementioned published paper [7]. MIF (migration inhibitory factor) in infiltrated macrophages was found to communicate with the extracellular domain of CD74, which is located in resident macrophages in both human and mouse kidneys. CD74 is required for MIF-induced activation of the extracellular signal–regulated kinase–1/2 MAP kinase cascade and cell proliferation. Previous research has shown that MIF-treated macrophages show greater phagocytic activity and can thus destroy more intracellular pathogens [39, 40]. Another shared signal transduction axis in human and mouse comprises CD47 and SIRPα. CD47 can bind to the SIRPα transmembrane protein on myeloid cells (especially macrophages) [41]. The extracellular IgV domain of SIRPα binds to CD47, which facilitates the release of “do not eat me” signals, which inhibit macrophage-mediated phagocytosis [42, 43]. Taken together, the data from our studies indicate that CD47 is needed infiltrated macrophages protection against damaged caused by binding to SIRPα located in resident macrophages in both human and mouse.

Each signaling molecule involved in cell–cell communication is functional only in resident macrophages (green edge, e.g., CD74), in infiltrated macrophages (orange edge, e.g., CCL3) or in both types of macrophages (yellow gray edge, e.g., CD47) when both species are taken into consideration (Fig. 6), showing the complexity of signaling transduction in terms of both species and cellular location.

## Discussion

RNA-seq data provide great advantages in terms of both biological status and genome coverage for the comprehensive understanding of transcriptome composition and diversity [44]. Because of its single-cell resolution and larger dynamic range, scRNA-Seq has recently become the preferred approach to gene expression profiling of different types of cells [45]. In this paper, we reanalyzed a set of scRNA-seq data generated by Zimmerman et al. [7] (10.1681/ASN.2018090931). The original analysis identified universal markers of renal resident macrophages in human, mouse, and rat. In this study, we characterized the evolutionarily conserved promoter architecture of renal macrophage. We found that gene expression variability across cell types and species shapes the relationship between renal resident and infiltrated macrophages. Genes with high transcriptional divergence in resident and infiltrated macrophages between species were associated with high transcriptional variability between cells. Genes with both high transcriptional divergence and variable across cells were found to be related to promoter sequences with enriched TATA-boxes and no CGIs.

DEGs encoding TFs might be under lower functional or regulatory constraints, owing to the versatility endowed by TF expression divergence among species. This condition may also be the reason that the authors who identified universal markers for renal resident macrophages among species [7] did not identify universal markers of infiltrated macrophages among species, although they sequenced all types of innate immune cells in kidneys.

Our findings suggest that the gene regulatory network plays critical roles in driving conserved expression and variation between renal resident and infiltrated macrophages in both human and mouse. Regulons that were correlated with at least one other regulon (|r|> 0.3) tended to be specifically active in resident or infiltrated macrophages, while other regulons tended to be simultaneously active in both kinds of macrophages. FOS and JUN are among 18 common regulon pairs found in human and mouse. Both JUN and FOS are regulated by the MAPK pathway, which was found to be enriched with upregulated DEGs in infiltrated macrophages in all species analyzed in our study. The FOS gene family consists of 4 members: FOS, FOSB, FOSL1, and FOSL2. The JUN gene family consists of 3 members: JUN, JUNB, and JUND. The genes in the FOS gene family encode leucine zipper proteins that can dimerize with proteins in the JUN family, thereby forming the transcription factor complex AP-1, which is related to cell proliferation and differentiation and the regulation of cytokines and growth factors [46]. Intriguingly, FOS and JUN, only this form of combination of AP-1 co-exists and shows a high correlation in human and mouse, which may be an essential mechanism in evolutionary conservation.

In addition, the transcriptional properties of renal macrophages in human and mouse were found to be maintained by distinct gene regulatory networks. A pySCENIC analysis revealed that TFs, which regulated > 80% of the DEGs, differed between the two species. This distinction was consistent with the difference in the top 5 DEGs in resident and infiltrated macrophages among different species, as shown in Fig. 1C in [7].

Using single-cell transcriptomics, we mapped promoter architecture during homeostasis. Transcriptionally divergent genes, such as those that encode TFs, varied across cells and exhibited distinct promoter structures. As shown in Fig. 5D, boundaries of cells with different labels in both species and macrophage subtype levels were explicitly classified, implying that the mechanism of transcriptional regulation is specific to renal macrophages from different species and that different subtypes reflect this specificity. Furthermore, the gene regulatory network of infiltrated macrophages between species showed comparatively better species-wide consistency than resident macrophages. The relatively conserved transcriptional gene regulatory network in infiltrated macrophages between species is presumably uniquely enriched in pathways related to kinases. The cell clusters based on gene expression and the gene regulatory network data were consistent. However, in some cases, the outcome of fuzzy clustering performed in the context of an intermediate state can be inaccurate. Signal transduction is complex and differs both with respect to species and cellular location. Future investigations are needed to verify the results in this paper, which were obtained in silico.

## Conclusions

Altogether, our work shows that transcriptionally divergent genes, such as the differentially TF-encoding genes expressed in resident and infiltrated macrophages across the three species, vary among cells and include distinct promoter structures. The gene regulatory network in infiltrated macrophages shows comparatively better species-wide consistency than resident macrophages. The conserved transcriptional gene regulatory network in infiltrated macrophages among species is uniquely enriched in pathways related to kinases, and TFs associated with largely conserved regulons among species are uniquely enriched in kinase-related pathways. Our work reveals that gene expression variability across cells and species shapes the relationship between renal resident macrophages and infiltrated macrophages.

## Methods

### Single-cell sequencing data processing

We obtained pre-processed scRNA-seq data from the Gene Expression Omnibus (GEO) database, www.ncbi.nlm.nih.gov/geo (accession No. GSE128993). We followed the vignette in the R package Seurat (version 3.2.0, https://satijalab.org/seurat/pbmc3k_tutorial.html) [47] to create the Seurat data matrix object. In brief, we retained all genes expressed in more than three cells and cells with at least 200 detected genes. Cells with mitochondrial gene percentages > 5% and unique gene counts > 2500 or < 200 were discarded.

The data were then processed using the "SCTransform" function in the R package Seurat. The first 100 principal components were selected as inputs for t-SNE using the functions "FindClusters" and "RunTSNE" in Seurat. Through a SCTransform assay, 13,425, 12,323, and 11,757 genes were retained for human, mouse, and rat cells, respectively. Resolutions of 0.2, 0.2, and 0.4 for FindClusters were used for human, mouse, and rat, respectively.

### Cell-specific gene set scoring analysis and pathway enrichment analysis

Cell-specific gene set scoring was analyzed for resident and infiltrated macrophages in kidney using the KEGG collection [48] in the Molecular Signatures Database (MSigDB) [49] (category, C2; subcategory, CP: KEGG) according to the vignette in the R package VAM (version 0.4.0, http://www.dartmouth.edu/~hrfrost/VAM/VAM_PBMC3K_SCTransform.pdf) [50]. The input is the processed result obtained through the "SCTransform" function. Enriched pathways with FDR-adjusted *P-*value < 0.05 were considered to be significant.

We explored the KEGG pathways enriched with DEGs using WebGestalt 2019 [51]. The enriched pathways with FDR-adjusted *P-*value < 0.05 were considered to be significant.

### Quantifying gene expression divergence between resident and infiltrated macrophages in kidney

Differential expression (fold change) for each gene between resident and infiltrated macrophages in the kidneys of all species was determined using Seurat's "FindMarkers" function (min.pct, 0.1; logfc.threshold, 0.25). Genes with FDR-adjusted *P-*values < 0.05 in the output file were considered to be differentially expressed. We calculated the differences between the fold-change (FC) estimates across orthologs to measure transcriptional divergence as follows:

$${\text{log}}\left[ {1/2 \times \sum\limits_{j} {\left( {{\text{log}}\left[ {\text{FC human}} \right] - {\text{log}}\left[ {{\text{FC rodent}}_{{\text{j}}} } \right]} \right)^{2} } } \right]$$

where rodents represent mouse and rat. We classified the 636 DEGs with one-to-one orthologues between resident and infiltrated macrophages in the kidney across all three species into three groups on the basis of their levels of transcriptional divergence: (1) genes with highly divergent expression between resident and infiltrated macrophages (the top 25% of genes with the highest divergence values across the three studied species); (2) low-divergence genes (the bottom 25% of genes with the least divergence); and (3) medium-divergence genes (50% of genes in the intermediate range).

To consider differences between species, we focus on between-clade differences (human versus rodents) rather than on within-clade differences. In this way, we mapped the most significant macroevolutionary differences along the longest branches of our three-species phylogeny. In addition, averaging within clades reduces noise [52]. Then, we can tell the difference of genes with different evolutionary consistency measured by fold changes between infiltrated and resident macrophages in each species.

We constructed a phylogenetic tree based on the change in gene expression between resident and infiltrated macrophages; all these genes with one-to-one orthologs across all three species and were expressed in at least one of these species were identified (Fig. 1D). We performed hierarchical clustering following the detailed procedures established in [35].

### Promoter sequence analysis

phastCons [53] values were used to assess promoter sequence conservation. We downloaded the base-by-base phastCons scores from the alignments of 6 vertebrate genomes with human in the UCSC (http://genome.ucsc.edu/) [54]. The phastCons scores were interpreted as probabilities of conservation of each base on the basis of the assumptions of the model and the maximum-likelihood parameter estimates.

A mean phastCons score for each of the 500 bp upstream of the TSS in the relevant human gene was calculated separately for the group of genes with high, medium and low transcriptional divergence, respectively. To plot the mean values of the three sets of divergent genes, the geom_smooth function in the R package ggplot2 was used with default parameters (with loess used as the smoothing method to depict the 95% confidence interval for the predictions based on a linear model). CGI and TATA-box annotations were defined as in [35]. TATA-box matches and CGI overlapping areas were both computed with respect to the TSS of the human genes.

### Cell-to-cell variability analysis

We applied the DM (distance to median) approach to quantify the biological cell-to-cell variability of each gene in resident and infiltrated macrophages in kidney for each species. DM is an established method used to calculate the cell-to-cell variability in gene expression while accounting for confounding factors, including the gene expression level [55]. This was performed with the pipeline listed in the vignette in the "DM" function in the R package scran, as also described in [55]. The input was the raw count matrix of resident and infiltrated macrophages in the kidney for each species.

### Comparison of transcriptional divergence between different functional groups

We categories the 636 DEGs with transcriptional divergence into the following functional groups: cytokines, chemokines and their receptors (GO: 0005125 (cytokine activity), GO: 0008009 (chemokine activity), GO: 0004896 (cytokine receptor activity), and GO: 0004950 (chemokine receptor activity)); TFs (as in TF classification (TFClass) [56]); kinases and phosphatases (GO: 0004672 (protein kinase activity) and GO: 0004721 (phosphoprotein phosphatase activity)).

The divergence values of these functional subsets were compared to the entire group of 636 DEGs. Gene lists belonging to the aforementioned GO annotations were downloaded using the highly customizable BioMart data mining tool [57] implanted in Ensembl (http://www.ensembl.org/biomart/martview) [58]. We mapped human TFs found by TFClass to the orthologs in mouse and rat using the BioMart data mining tool.

### Gene regulatory network analysis

The gene regulatory networks of renal macrophages in human and mouse were inferred using the Python package pySCENIC (single-cell regulatory network inference and clustering, version 1.1.3) [30]. The pySCENIC analysis consisted of three steps: i) construction of a co-expression network using GENIE3 [59], ii) identification of direct binding by DNA-motif analysis using RcisTarget, and iii) inference of activity of regulons using AUCell [60]. The details are as follows:

(i) The log-normalized gene expression matrix of macrophages in human and mouse generated using Seurat was used as input data, respectively. The gene sets coexpressed with TFs were identified using GENIE3.
(ii) After running GENIE3, motif datasets (mm10__refseq-r80__500bp_up_and_100bp_down_tss.mc9nr.feather, mm10__refseq-r80__10kb_up_and_down_tss.mc9nr.feather; hg38__refseq-r80__500bp_up_and_100bp_down_tss.mc9nr.feather, hg38__refseq-r80__10kb_up_and_down_tss.mc9nr.feather) were used to construct regulons of the human and mouse by RcisTarget, respectively. The regulons were based on the known TF-targets and motif information catalogued in RcisTarget database.
(iii) Finally, the activity values of regulons were inferred with gene matrix by using AUCell according to its online pipeline step-by-step (https://rawcdn.githack.com/aertslab/SCENIC/0a4c96ed8d930edd8868f07428090f9dae264705/inst/doc/SCENIC_Running.html). Regulon activity was a binarized score [0 for inactive and 1 for active] and was assigned to each regulon in each cell. The regulon activity can be achieved with expression matrix and regulons from the same species (e.g. Fig.4A, D), or from different species by converting TFs and the corresponding target genes into the other species (e.g. Fig.4B, C).

Selectivity is an established measure [61]. The selectivity score for each TF in human and mouse was calculated as follows:

$${\text{Selectivity score }} = \frac{{\# {\text{DEG}} \cap \# {\text{target gene}}}}{{\# {\text{target gene}}}}$$

where DEGs were the 636 DEGs between resident and infiltrated macrophages in at least one of the studied species (human, mouse, and rat) and with one-to-one orthologs across the studied species; target genes are regulated by a certain TF. Cumulative plots illustrating the coverage of DEGs by the TFs were generated by using TFs sorted on the basis of the selectivity score in descending order.

### Cross-species regulon comparisons

The TFs in the mouse regulon binary matrix (achieved by pySCENIC) were converted into homologous human TFs. A total of 161 TFs overlapped in human and mouse regulon binary matrices were retained. The cells were clustered based on the merged binary activity matrix using Ward's hierarchical clustering with Spearman's distance.

To establish an alternative approach based only on expression, we generated a merged expression matrix where mouse genes were also converted into homologous human genes (only the genes available in both matrices were retained). Each matrix was normalized by Z score for each gene before merging.

### Normalized binary entropy

Since there were only two types of cells (resident and infiltrated macrophages) in our study, we used a normalized binary entropy score to quantify the regulon specificity for the two subtypes of macrophages. First, the percentage of active cells of a regulon in resident macrophages was normalized by dividing the sum of the percentage of active cells in both resident and infiltrated macrophages. Then, the sum of the normalized percentage of resident and infiltrated macrophages was set equal to 1. Second, the normalized binary entropy score of each regulon was calculated as

$${\text{Normalized binary entropy score }} = \, - {\text{p }} \times {\text{ logp }} - \, \left( {{1 } - {\text{ p}}} \right) \, \times {\text{ log}}\left( {{1 } - {\text{ p}}} \right)$$

where p was the normalized percentage of the active resident macrophages of a certain regulon. The entropy score ranged from 0 to 1. A higher entropy score indicated a higher level of uncertainty of the subtype of macrophage (resident or infiltrated) to which the regulon belonged [62].

## Supplementary Information

**Additional file 1**. Supplementary Legends for Supplementary Figures. **Figure S1**. The classification system of transcriptional divergence and the corresponding divergence values. **Figure S2**. Distribution of divergence values of different categories of DEGs. **Figure S3**. Cell-to-cell variability estimation using DM and mean expression levels. Cell-to-cell variability (as estimated by the DM (distance from median) method) versus mean expression level, measured for n = 636 DEGs in 318 resident macrophages (left) and 143 infiltrated macrophages (right) in human kidney. Expression levels are binned into 5 equal-sized groups (127 genes in a group). **Figure S4**. Joint clustering of human and mouse renal scRNA-seq data based on expression (A) and regulon activity (B). **Figure S5**. Heatmaps of mouse sepsis kidney samples at 48h clustered by the activity of all regulons which are extracted from their own expression data. Active regulons per cell appear in black; the horizontal color bar indicates the corresponding subset of each cell. **Figure S6**. Bi-clustering of macrophages showing 173 and 95 regulons which are correlated with at least one other regulon (|r| > 0.3) in human (A) and in mouse (B), respectively. Active regulons per cell appear in black; the horizontal color bar indicates the subset associated with each cell.

**Additional file 2**. Supplementary Tables. **Table S1**. DEGs between resident and infiltrated macrophages in human. **Table S2**. DEGs between resident and infiltrated macrophages in mouse. **Table S3**. DEGs between resident and infiltrated macrophages in rat. **Table S4**. Enriched pathways of DEGs upregulated in resident macrophages. **Table S5**. Enriched pathways of DEGs upregulated in infiltrated macrophages. **Table S6**. DEGs between resident and infiltrated macrophages in at least one of the studied species (human, mouse, and rat) and with one-to-one orthologs across the studied species.  The DEGs are shown with human gene symbols. **Table S7**. Regulons and the corresponding attributes in renal macrophages in human. **Table S8**. Regulons and the corresponding attributes in renal macrophages in mouse. **Table S9**. KEGG enrichment of TFs which belong to regulons with stronger evolutional conservation in infiltrated macrophages. **Table S10**. KEGG enrichment of TFs which belong to regulons with weaker evolutional conservation in infiltrated macrophages. **Table S11**. GO enrichment of TFs belonging to regulons which are preferably active in C4C6 branch. **Table S12**. GO enrichment of TFs belonging to regulons which are preferably active in C0 branch. **Table S13**. Cellular crosstalk between resident and infiltrated macrophages in human. **Table S14**. Cellular crosstalk between resident and infiltrated macrophages in mouse. **Table S15**. Common regulon pairs in human and mouse where regulons show comparatively high correlation with each other (|r| > 0.3)

## Data Availability

The datasets generated or analysed during this study are available in the Gene Expression Omnibus (GEO) repository, GSE128993 and GSE151658.
