# Supplementary material for: Gene expression variability across cells and species shapes the relationship between renal resident macrophages and infiltrated macrophages
Source: BMC Bioinformatics. 2023 Mar 1;24:72. doi: 10.1186/s12859-023-05198-z (PMC9976410; doi:10.1186/s12859-023-05198-z)

**Supplementary Figures**

**Figure S1** The classification system of transcriptional divergence and the corresponding divergence values.


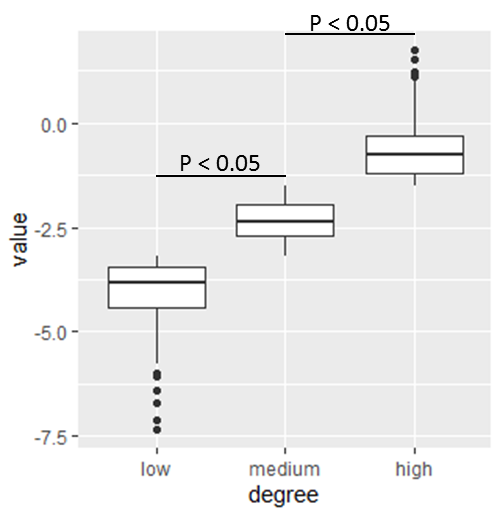


**Figure S2** Distribution of divergence values of different categories of DEGs.


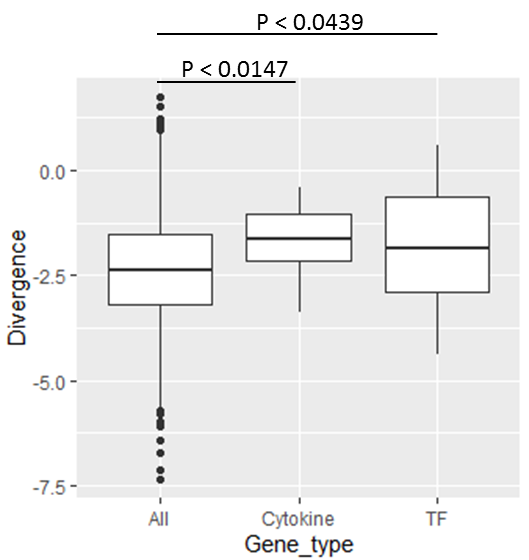


**Figure S3** Cell-to-cell variability estimation using DM and mean expression levels. Cell-to-cell variability (as estimated by the DM (distance from median) method) versus mean expression level, measured for n = 636 DEGs in 318 resident macrophages (left) and 143 infiltrated macrophages (right) in human kidney. Expression levels are binned into 5 equal-sized groups (127 genes in a group).


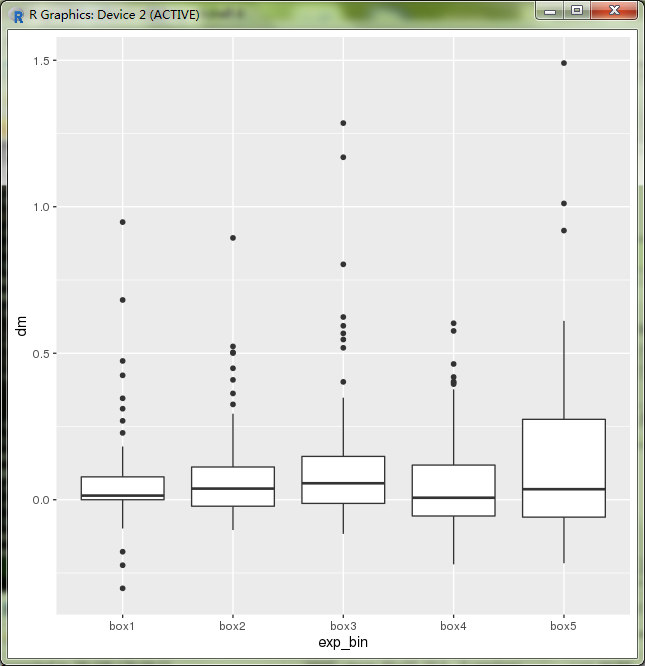

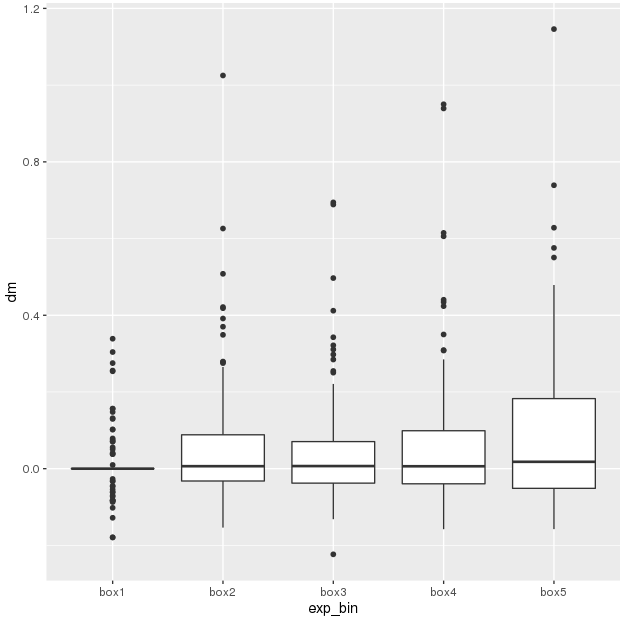


**Figure S4** Joint clustering of human and mouse renal scRNA-seq data based on expression (A) and regulon activity (B).

1.
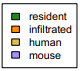
 (B)


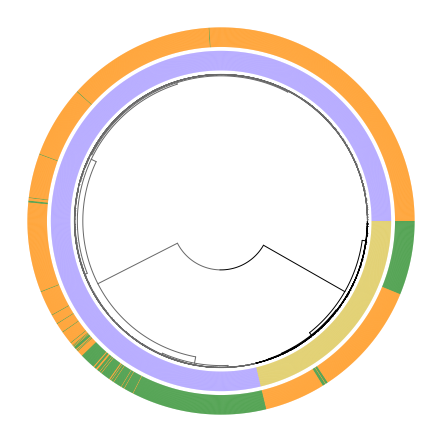

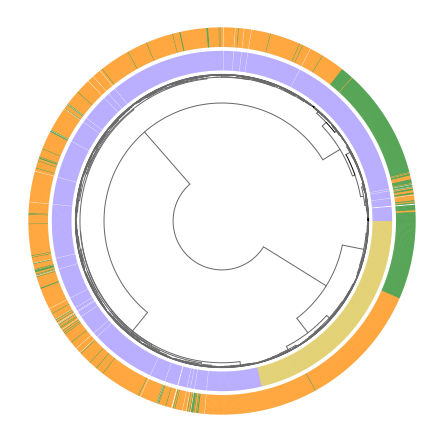


**Figure S5** Heatmaps of mouse sepsis kidney samples at 48h clustered by the activity of all regulons which are extracted from their own expression data. Active regulons per cell appear in black; the horizontal color bar indicates the corresponding subset of each cell.

A


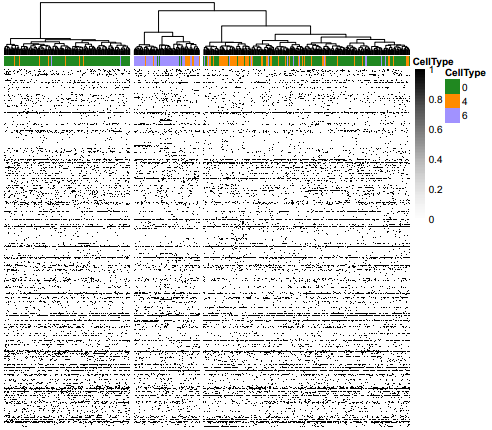


**Figure S6** Bi-clustering of macrophages showing 173 and 95 regulons which are correlated with at least one other regulon (|r| > 0.3) in human (A) and in mouse (B), respectively. Active regulons per cell appear in black; the horizontal color bar indicates the subset associated with each cell.

A


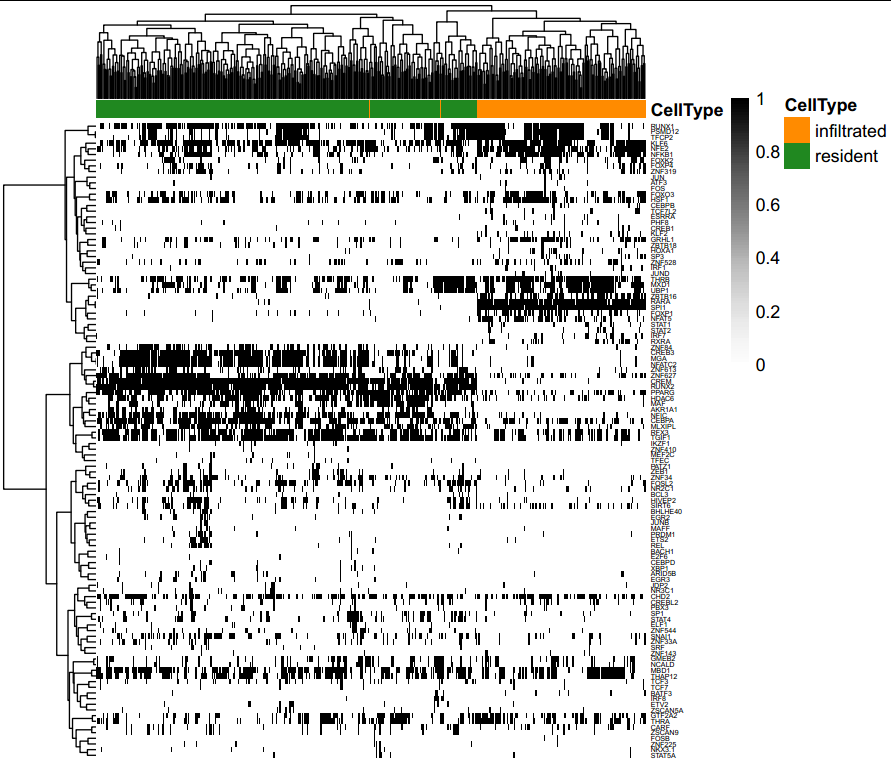


B


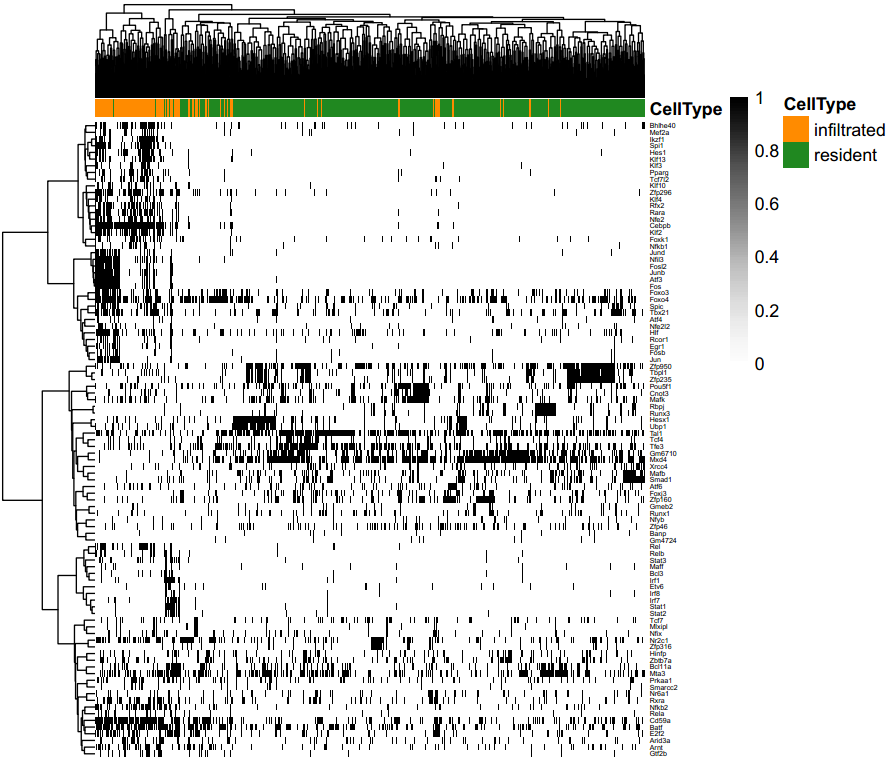

Supplement: Supplementary file 1 — Additional file 1. Supplementary Legends for Supplementary Figures. Figure S1. The classification system of transcriptional divergence and the corresponding divergence values. Figure S2. Distribution of divergence values of different categories of DEGs. Figure S3. Cell-to-cell variability estimation using DM and mean expression levels. Cell-to-cell variability (as estimated by the DM (distance from median) method) versus mean expression level, measured for n = 636 DEGs in 318 resident macrophages (left) and 143 infiltrated macrophages (right) in human kidney. Expression levels are binned into 5 equal-sized groups (127 genes in a group). Figure S4. Joint clustering of human and mouse renal scRNA-seq data based on expression (A) and regulon activity (B). Figure S5. Heatmaps of mouse sepsis kidney samples at 48h clustered by the activity of all regulons which are extracted from their own expression data. Active regulons per cell appear in black; the horizontal color bar indicates the corresponding subset of each cell. Figure S6. Bi-clustering of macrophages showing 173 and 95 regulons which are correlated with at least one other regulon (|r| > 0.3) in human (A) and in mouse (B), respectively. Active regulons per cell appear in black; the horizontal color bar indicates the subset associated with each cell. [file 12859_2023_5198_MOESM1_ESM.docx]
